# Insights into the sand fly saliva: Blood-feeding and immune interactions between sand flies, hosts, and *Leishmania*

**DOI:** 10.1371/journal.pntd.0005600

**Published:** 2017-07-13

**Authors:** Tereza Lestinova, Iva Rohousova, Michal Sima, Camila I. de Oliveira, Petr Volf

**Affiliations:** 1 Department of Parasitology, Faculty of Science, Charles University, Prague, Czech Republic; 2 Instituto Gonçalo Moniz FIOCRUZ, Salvador, Brazil; Institut Pasteur, FRANCE

## Abstract

**Background:**

Leishmaniases are parasitic diseases present worldwide that are transmitted to the vertebrate host by the bite of an infected sand fly during a blood feeding. Phlebotomine sand flies inoculate into the mammalian host *Leishmania* parasites embedded in promastigote secretory gel (PSG) with saliva, which is composed of a diverse group of molecules with pharmacological and immunomodulatory properties.

**Methods and findings:**

In this review, we focus on 3 main aspects of sand fly salivary molecules: (1) structure and composition of salivary glands, including the properties of salivary molecules related to hemostasis and blood feeding, (2) immunomodulatory properties of salivary molecules and the diverse impacts of these molecules on leishmaniasis, ranging from disease exacerbation to vaccine development, and (3) use of salivary molecules for field applications, including monitoring host exposure to sand flies and the risk of *Leishmania* transmission. Studies showed interesting differences between salivary proteins of *Phlebotomus* and *Lutzomyia* species, however, no data were ever published on salivary proteins of *Sergentomyia* species.

**Conclusions:**

In the last 15 years, numerous studies have characterized sand fly salivary proteins and, in parallel, have addressed the impact of such molecules on the biology of the host–sand fly–parasite interaction. The results obtained shall pave the way for the development of field-application tools that could contribute to the management of leishmaniasis in endemic areas.

## Background

Phlebotomine sand flies (Diptera: Phlebotominae) are blood-feeding insects of medical and veterinary importance that transmit parasites from the genus *Leishmania* (Kinetoplastida: Trypanosomatidae). These protozoan parasites are the causative agents of leishmaniases, neglected infectious diseases that affect people in 98 countries. They are manifested by different clinical symptoms ranging from the disfiguring cutaneous and muconasal form to the fatal visceral form, if left untreated. The outcome of infection is influenced by the virulence of the parasite strain but also by the host’s genetic background and immune status (reviewed in [1, 2]). The annual incidence was estimated to be approximately 0.2–0.4 million and 0.7–1.2 million cases for visceral and cutaneous leishmaniasis, respectively. This burden ranks leishmaniasis to the ninth place of all human infectious diseases, e.g., [3, 4].

The metacyclic promastigotes—the infectious form of *Leishmania* embedded in promastigote secretory gel (PSG) (reviewed in [5])—are transmitted to the vertebrate hosts by the bites of female sand flies from the genus *Phlebotomus* in the Old World or *Lutzomyia* in the New World (reviewed in [6]). In the gut of the invertebrate vector, *Leishmania* parasites occur in several morphological forms of extracellular flagellated promastigotes (reviewed in [7]), while in vertebrate hosts, they occur as immobile amastigotes inside parasitophorous vacuoles in phagocytic cells, mainly macrophages. Macrophages are able to kill or to long-term host intracellular forms of *Leishmania* sp. depending on their state of activation. While inflammation-promoting "classically activated" macrophages produce nitric oxide and other toxic intermediates resulting in the destruction of *Leishmania* parasites, anti-inflammatory "alternatively activated" macrophages tend to the production of urea and L-ornithine. The latter is a building element for synthesis of polyamines, which are beneficial for *Leishmania* intramacrophage growth (reviewed in [8–10]).

The success of infection by *Leishmania* parasites is a result of a long host–parasite coevolutionary process and it is linked with the ability of the parasite to manipulate the vertebrate host immune system in its favor. Affecting the host immune response occurs not only by means of molecules produced by parasites but also by vector saliva molecules, which are obligately injected into the blood-feeding site during transmission as well as during noninfectious feeding.

## Sand fly salivary glands structure and composition

**Fig 1:**
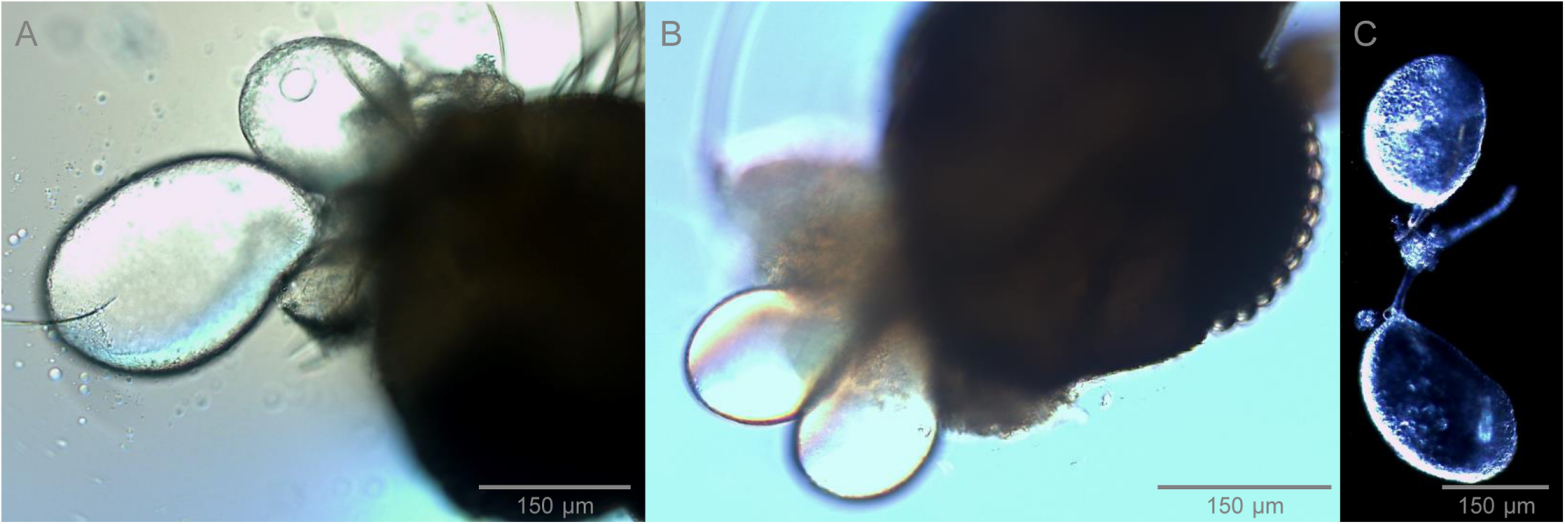
Salivary glands of sand flies: Comparison of morphologically heterogeneous and homogeneous glands. **(A)** Pair of fully inflated heterogeneous glands of *Phlebotomus duboscqi* (measurring 269 x 178 μm and 187 x 138 μm). **(B)** Pair of fully inflated homogeneous glands of *Lutzomyia longipalpis* (measurring 166 x 106 μm and 168 x 104 μm). Nomarski interference contrast (A, B) and dark-field microscopy for *P*. *duboscqi* salivary glands **(C)** were used. Glands were measured by Image-J software.

Sand fly salivary apparatus consists of 2 salivary glands, ducts, a pump, and a channel (Fig 1C). Salivary glands are a paired, hollow organ surrounded by a single layer of epithelium. The glands can be heterogeneous or homogeneous in terms of size and shape, depending on the sand fly species [11]. For example, the bigger, fully inflated gland of *P*. *papatasi* may reach about 190 x 160 μm, while a smaller one is about 165 x 140 μm [11, 12]. Similar morphological heterogeneity can be found in *P*. *duboscqi* (Fig 1A) and seems to be typical for members of subgenus *Phlebotomus*, as all other sand flies studied (members of *Phlebotomus* subgenera *Larroussius*, *Adlerius*, *Paraphlebotomus*, and *Euphlebotomus* and genus *Lutzomyia*) possess a morphologically homogeneous pair of salivary glands (Fig 1B).

The composition of sand fly saliva differs not only among different species [13, 14] but the difference can be sometimes detected also among populations originating from distinct geographical areas [13, 15–18]. The protein content of saliva differs among species and colonies used, condition of their maintenance, and by sensitivity of methods for protein-concentration measurement [19], however, protein concentrations range approximately from 0.18 to 0.8 μg/gland [12, 19]. An important difference is evident between blood-feeding females and nonhematophagous males; the concentration of salivary proteins from *P*. *duboscqi* saliva was almost 30 times higher in case of females compared with males [13]. The number of bands in salivary gland homogenate (SGH) revealed by SDS-PAGE also differed considerably between genders; in females, 8 major bands were detected, whereas just 1 was observed in males [13]. Concurrently, the number of salivary proteins is correlated with the female age when a complete SDS-PAGE salivary profile has been achieved (on days 3 and 5 in females maintained at 26°C and room temperature, respectively) [13, 20].

For better identification and characterization of salivary proteins, it is advantageous to know the nucleotide and amino acid sequences of these proteins. The initial characterization of sand fly salivary proteins started in 1999, when Charlab et al. identified several proteins in *Lutzomyia longipalpis* saliva by cloning combined with biochemical approaches [21]. The complete cDNA library of salivary glands of this *Leishmania infantum chagasi* vector was obtained 5 years later when *Lu*. *longipalpis* salivary proteins were identified by cDNA sequencing, proteomics, and customized computational biology approaches [22]. Meanwhile, transcriptomic analysis of salivary proteins of *P*. *papatasi*, the *L*. *major* vector, was published [23] and updated later [24]. Up to date, approximately 800 sand fly species are known worldwide and less than 100 are suspected or proven *Leishmania* vectors (reviewed in [6]). However, salivary cDNA libraries from only 13 sand fly species have been constructed: for 9 species of the genus *Phlebotomus* and 4 species of the genus *Lutzomyia* (Table 1).

**Table 1:** Sand fly species with published salivary glands–cDNA libraries.

Genus	Subgenus	Species	Reference
*Phlebotomus*	*Phlebotomus*	*Phlebotomus papatasi*	Valenzuela et al. 2001 [23], Abdeladhim et al. 2012 [24]
		*P*. *duboscqi*	Kato et al. 2006 [35]
	*Paraphlebotomus*	*P*. *sergenti*	Rohousova et al. 2012 [36]
	*Euphlebotomus*	*P*. *argentipes*	Anderson et al. 2006 [33]
	*Adlerius*	*P*. *arabicus*	Hostomska et al. 2009 [34]
	*Larroussius*	*P*. *perniciosus*	Anderson et al. 2006 [33], Martín-Martín et al. 2013 [157]
		*P*. *ariasi*	Oliveira et al. 2006 [37]
		*P*. *tobbi*	Rohousova et al. 2012 [36]
		*P*. *orientalis*	Vlkova et al. 2014 [32]
*Lutzomyia*	*Lutzomyia*	*Lutzomyia longipalpis*	Charlab et al. 1999 [21], Valenzuela et al. 2004 [22]
	*Helcocyrtomyia*	*Lu*. *ayacuchensis*	Kato et al. 2013 [39]
	*Nyssomyia*	*Lu*. *intermedia*	de Moura et al. 2013 [38]
		*Lu*. *olmeca*	Abdeladhim et al. 2016 [25]

More than 20 diverse proteins belonging to the different protein families have been identified in each cDNA library. Several of these families are shared among all tested species usually containing more than 1 homologue. Protein families that were detected in selected *Phlebotomus* as well as in *Lutzomyia* species are: antigen 5–related proteins, apyrases, odorant-binding proteins (D7-related proteins and PpSP15-like proteins), yellow-related proteins (YRPs), silk-related proteins, and lufaxin-like proteins [25].

For purpose of this review, only the major protein families will be further discussed in detail concerning their biological functions and antigenic properties.

## Properties of sand fly saliva

During the process of taking a blood meal, the skin of vertebrate hosts is damaged by the proboscis of sand flies. The host fights back by means of 3 effective systems, hemostasis, inflammation, and immunity, which hinder the successful feeding of the insect. Sand fly saliva is composed of pharmacologically active components called sialogenins with antihemostatic, anti-inflammatory, and immunomodulatory properties, which help to circumvent this inhospitable host environment and to successfully finish the blood meal (reviewed in [26, 27]).

### Saliva in hemostasis and blood feeding

Hemostasis is a physiological process by which the hosts can control the loss of blood after injury, including insect bite. It consists of 3 phenomena: platelet aggregation, blood coagulation, and vasoconstriction, which form the first major barriers for sand flies to successfully obtain blood (reviewed in [27, 28]). Sand flies circumvent this feeding problem by producing various salivary components that counteract the host’s hemostatic system.

The most common enzyme confirmed in several blood-sucking arthopods (reviewed in [26]) is an apyrase, which hydrolyzes nucleotide triphosphates (ATP) and diphosphates (ADP) to a monophosphate (AMP) and an inorganic phosphate (Pi). This hydrolytic activity prevents the platelet aggregation that is normally induced by ADP released from damaged cells and activated platelets at the feeding site. Three classes of apyrases have already been characterized: "5´-nucleotidase family," isolated for the first time from salivary glands of *Aedes aegypti* [29]; "CD 39 family of nucleotidases," isolated from flea *Xenopsylla cheopis* [30]; and "Cimex family," strictly calcium dependent, originally identified in the bedbug *Cimex lectularius* [31], later discovered in sand flies *P*. *papatasi* [23] and *Lu*. *longipalpis* [21]. To date, "Cimex family" of apyrases was found also in other sand fly species studied: *P*. *orientalis* [32], *P*. *argentipes* and *P*. *perniciosus* [33], *P*. *arabicus* [34], *P*. *duboscqi* [35], *P*. *sergenti* and *P*. *tobbi* [36], *P*. *ariasi* [37], *Lu*. *intermedia* [38], *Lu*. *ayacuchensis* [39], and *Lu*. *olmeca* [25]. Sand fly apyrases are proteins with molecular mass varying approximately from 33 kDa–36 kDa. In individual species, they occur mostly in 1 homologue, but in several species, 2 (*P*. *perniciosus*, *P*. *duboscqi*, and *P*. *tobbi*) or 3 (*P*. *arabicus*, *P sergenti*, and *P*. *orientalis*) apyrases were detected [32–36].

Another plentiful family of salivary proteins occurring in sand flies is a group of odorant-binding proteins belonging to the bigger group of proteins containing pheromone-binding proteins and general odorant-binding proteins. In sand fly saliva, it is represented with 2 groups of proteins: PpSP15-like proteins and D7-related proteins (reviewed in [27]). The function of D7-related proteins (with molecular mass about 27 kDa) in sand fly saliva remains unclear, but similar proteins in mosquitoes are proven binders of biogenic amines or eicosanoids [40, 41] and play a role as anticoagulants [42, 43]. PpSP15-like proteins have approximately 15 kDa and are sand fly specific with very abundant and highly variable amino acid sequences [33, 34, 36, 39], which could result in different functions among individual sand fly species. Two SP15-like proteins isolated from *P*. *duboscqi* (SP15a and SP15b) bind with high affinity to the negatively charged surface of polymers including polyphosphate, heparin, and dextran sulfate, whereby they compete for the binding sites with coagulation factor XII and inhibit coagulation [44].

In *Lu*. *longipalpis*, an anticoagulant named Lufaxin was recently described [45]. It is a potent inhibitor of factor Xa, which normally plays a key role in the coagulation cascade leading to trombin production and fibrin clot formation. The blockage of this factor prevents blood coagulation in the feeding site [45]. Homologues of Lufaxin were found in all sand flies studied so far (Table 1) but their function has not yet been confirmed.

One of the key mechanisms for successful blood feeding is removing biogenic amines from the feeding site, e.g., by binding them into the proteins commonly named as kratagonists. Binding of these small molecules (such as serotonin, histamine, and catecholamines) leads to prevention of inflammation and hemostasis, thus allowing the blood-feeding process (reviewed in [46]). Histamine is present in granules of mast cells and basophils, from which it can be released. Serotonin can be detected in human platelets, digestive tract, or the central nervous system (reviewed in [47]). Blocking those amines results in vasodilatation, platelet deactivation, and decreased vascular permeability [48, 49]. Arthropod salivary proteins with proven amine-binding function can be divided in 3 groups, (1) lipocalins, (2) D7 proteins, and (3) YRPs. The common feature of all 3 groups is their shape—hollow barrel with 2 possible entrances and the ligand-binding pocket inside of this structure [40, 41, 50–53]. In sand flies, 2 protein families can serve as putative kratagonists, D7-related proteins and YRPs, which are present in all sand fly species tested so far. Nevertheless, amine-binding ability was described only for YRPs [50]. Conversely, lipocalins have not been described in any of the 13 salivary transcriptomes of sand fly species. YRPs are highly conserved and have similar molecular mass, between 40 kDa–42 kDa. There is a high variability in number of YRPs among different sand fly species; for example, only 1 member of YRPs was found in *P*. *arabicus* [34], *P*. *argentipes* [33], and *Lu*. *intermedia* [38], but on the contrary, 5 YRPs were detected in *P*. *sergenti* [36]. This variability might be attributed to the sensitivity of sequencing method, however, it is not likely for those species/cDNA libraries that were constructed in the same laboratory under the same conditions [33, 36]). Thus, the occurrence of various numbers of YRPs in sand flies could be caused by differential gene expression, because genes with lower expression might be recorded less frequently, as it was proven in other sand fly protein families [17]. In 2011, Xu et al. expressed 3 recombinant YRPs of *Lu*. *longipalpis* in *Escherichia coli* system. They determined their binding abilities to 6 different biogenic amines (norepinephrine, epinephrine, serotonin, dopamine, octopamine, or histamine) and characterized a crystal structure of 1 of these YRPs (LJM11) [50]. They proved that there is 1 ligand-binding site in the protein structure and that all 3 YRPs bind 5 different biogenic amines with various affinities. The highest affinity was observed for serotonin with all 3 proteins and no detectable binding was discovered for histamine with LJM11 and LJM111 and for epinephrine with LJM17 [50]. Sequence analysis of the ligand-binding pocket revealed a highly conserved amino acid motif among other sand flies. In this pocket, 5 out of 11 binding amino acids were identical for all sand fly YRPs [54]. The experiments were performed with only 1 sand fly species so far, *Lu*. *longipalpis* [50]. However, based on high sequence conservancy and protein modeling, it was suggested that all YRPs are able to bind various biogenic amines with different affinities [54].

Another compound neutralizing the host’s hemostatic process, isolated from the salivary glands of *Lu*. *longipalpis*, is a vasodilator peptide named maxadilan, which promotes an increase in blood flow and facilitates feeding [55, 56]. The vasodilator similar to maxadilan was not found in Old World sand fly species, but in *P*. *papatas*i saliva, large amounts of purines 5´AMP and adenosine were revealed [57]. Adenosine was previously described as a strong platelet-aggregation inhibitor [58, 59], which increases the concentration of platelet cyclic AMP. Simultaneously, both substances (5´AMP and adenosine) are known for their vasodilatory functions [60].

Hyaluronidases and endonucleases belong among other commonly occurring salivary components. They are not directly associated with disruption of hemostasis but facilitate feeding (reviewed in [46]). Hyaluronidase is an enzyme that degrades hyaluronan and some other glycosaminoglycans occuring in the extracellular matrix of the host skin [61]. Enzymatic activity of hyaluronidases seems to be substantial for insects taking blood from superficial hemorrhagic pools, including sand flies. This enzyme is often called "spreading factor" because of the ability to decrease the skin matrix viscosity around the feeding site and hence easily spread other pharmacologically active compounds present in saliva [61]. To date, the enzymatic activity of hyaluronidases has been found in all tested *Phlebotomus* and *Lutzomyia* species [19, 21, 32, 34, 36, 61]. The release of host DNA and thereby the lowering of local viscosity is also caused by salivary endonucleases (reviewed in [46]), for example, by an endonuclease described from *Lu*. *longipalpis* [22] named Lundep. The catalytic activity of Lundep is responsible for the destroying of neutrophil extracellular traps, which normally promote thrombus organization and stability, and is also known for anticoagulant properties (inhibiting the activation of factor XIIa) [62].

The above-mentioned list of salivary components is not complete, but a more detailed description of functionally known sialogenins is beyond the scope of this review. More thorough overview of sand fly salivary cocktail is summarized, for example, in [27, 46].

### Immunomodulatory effects of sand fly saliva on macrophage functions

Apart from antihemostatic properties, sand fly saliva is chemotactic for different immune cells, thereby modifying inflammatory processes at the blood-feeding site. Although many cell types, including monocytes and dendritic cells, interact with *Leishmania* parasites, this review is focused on macrophages, in which parasites grow and divide preferentially, and on neutrophils (see Early phase of infection), which appear to be important especially at early phase of infection, when parasites are inoculated into the skin. Saliva of *P*. *papatasi*, *Lu*. *longipalpis*, *Lu*. *intermedia*, and *P*. *duboscqi* significantly enhanced positive chemotaxis for macrophages, thus accelerating the entry of parasites into these cells [63–67]. In an air-pouch model, Teixeira et al. observed macrophages influx after an addition of *Lu*. *longipalpis* saliva in BALB/c and C57BL/6 mice [66]. SGH of *Lu*. *longipalpis* induced a significant attraction of macrophages in BALB/c strain, directly correlating with the higher chemokine expression of CC chemokine ligand 2/monocyte chemoattractant protein-1 (CCL2/MCP-1). On the other hand, in C57BL/6 mice, expression of these chemokines was weakly induced. This means that the same salivary components can cause different inflammatory effects according to host background [66].

The effect of sand fly saliva on a variety of macrophage functions was examined in detail especially for Old World species *P*. *papatasi* and New World sand fly *Lu*. *longipalpis*. Hall and Titus described that saliva of *P*. *papatasi* inhibits the ability of interferon gamma (IFN-γ) to activate macrophages to the production of nitric oxide (NO); this inhibitory effect facilitates parasite survival [68]. Indeed, an addition of *P*. *papatasi* SGH to macrophages caused reduction in inducible nitric oxide synthase (iNOS) mRNA expression [69, 70]. Later, the small, ethanol-soluble salivary molecule resistant to boiling was defined to be responsible for this down-regulation of the iNOS gene expression and reduction of NO production through the inhibition of protein phosphatase 1 and protein phosphatase 2A [70], 2 phosphatases with key function in the signaling pathway leading to nitric oxide synthesis [71]. One year later, these phosphatase inhibitors were revealed as 5´AMP and adenosine [57]. The adenosine itself was able to reduce the iNOS gene expression to the same degree as *P*. *papatasi* saliva [72].

Moreover, the ability of *P*. *papatasi* saliva to decrease the secretion of pro-inflammatory cytokines and to enhance the production of anti-inflammatory cytokines, which modulate macrophage effector functions, was described [69, 73]. Salivary gland lysate of *P*. *papatasi* inhibited interleukin 12 (IL-12) and IFN-γ expression, while the expression of interleukin 4 (IL-4) cytokine, which may interfere with the development of a protective Th1 response, was up-regulated in mice [69]. The cellular immune response against the saliva of *P*. *papatasi* in humans naturally exposed to sand fly bites was characterized by high levels of interleukin 10 (IL-10), which inhibits proliferation of lymphocytes producing IFN-γ [74]. The polarization of immune response towards Th2 was also observed after addition of adenosine alone—the production of IL-12, IFN-γ, and tumor necrosis factor alpha (TNF-α) was decreased [75–77] while IL-10 was increased [78].

A similar effect of saliva on host immunity was also observed in the case of *Lu*. *longipalpis*. Saliva induced an increase in interleukin 6 (IL-6), interleukin 8 (IL-8), and interleukin 12p40 (IL-12p40) production but decreased TNF-α and IL-10 production by lipopolysaccharide-stimulated human monocytes [79]. On the contrary, increased level of IL-10 associated with decreased NO production was observed in bone marrow–derived macrophages exposed to *Lu*. *longipalpis* SGH [80]. Aforementioned observation confirms that genetic differences among hosts may influence the immune responses elicited by salivary proteins from the same sand fly species. In addition, maxadilan itself was described to modulate host immune response to a similar degree as the whole saliva [81]. Maxadilan up-regulates the cytokines associated with a type 2 response, such as IL-10, IL-6, and transforming growth factor beta (TGF-β), but down-regulates type 1 cytokines such as interleukin 12p70 (IL-12p70), IFN-γ and TNF-α [73, 82, 83].

Furthermore, *Lu*. *longipalpis* saliva was shown to induce lipid body formation and prostaglandin E_2_ (PGE_2_) production by peritoneal macrophages *ex vivo* and *in vitro* [84]. PGE_2_, an eicosanoid derived from arachidonic acid, is mostly produced in cytoplasmic organelles called lipid bodies, which are created in leukocytes and other cells in response to inflammatory stimuli (reviewed in [85]). Prostaglandins contribute to the development of an anti-inflammatory response and also have vasodilatory effect [84]. An increasing production of PGE_2_ by macrophages was also shown after addition of maxadilan alone [82].

## Effect of saliva on leishmaniasis

If a sand fly delivers *Leishmania* parasites, they will be coinoculated with saliva to the same blood-feeding site. Thereafter, parasites can benefit from this by means of vector saliva–altered site (reviewed in [27]).

### Early phase of infection

The above-mentioned chemotactic effect of saliva (see section Immunomodulatory effects of sand fly saliva on macrophage functions) was more pronounced when the *Leishmania* parasites were added to inoculum; the greater number of recruited neutrophils and macrophages was observed [66]. The phagocytes influx was beneficial to parasites because of their early entry into these cells. Promastigotes that fail to get internalized into the professional phagocytes are rapidly degraded by cytotoxic activity of natural killer cells, neutrophils, and eosinophils in the vertebrate host [86]. Therefore, it is essential for promastigotes to invade macrophages as quickly as possible.

The importance of neutrophils as the first-recruited host cells to the feeding site and for the pathogen entry was confirmed by Peters et al. in 2008 [87]. *Leishmania* can survive temporarily inside neutrophils, which protect parasites from the hostile extracellular host environment (reviewed in [88]). The sand fly saliva alone or in combination with *Leishmania* parasites was described as a robust stimulus for an accumulation of neutrophils at the inoculation site in murine or hamster models [66, 87, 89–91]. Moreover, Prates et al. showed that salivary gland sonicate (SGS) of *Lu*. *longipalpis* enhances caspase-dependent and Fas ligand-mediated neutrophil apoptosis associated with enhanced *Leishmania* survival inside these cells. At the same time, neutrophils incubated with *L*. *chagasi* plus SGS produced significantly higher amounts of MCP-1 (CCL2), a chemokine that attracts numbers of macrophages for clearance of these recruited infected neutrophils [92, 93]. Van Zandbergen et al. suggested that infected apoptotic neutrophils can serve as "Trojan horses" to transfer *Leishmania* silently to macrophages inside apoptotic neutrophils [92]. Later, the "Trojan rabbit" hypothesis was admitted; in this scenario, *Leishmania* parasites escaped from dying neutrophils before ingestion by macrophages [87, 88].

### Enhancing effect of saliva

The effect when sand fly saliva exacerbates the infection caused by *Leishmania* sp. is called "enhancing effect." *L*. *major* coinjected with *Lu*. *longipalpis* or *P*. *papatasi* saliva resulted in a more severe disease reflected by a larger lesion when compared with a group of mice receiving parasites alone [94, 95]. Coinoculation of *P*. *papatasi* SGH with *L*. *major* even converted the naturally resistant mouse strain (C57BL/6) into a nonhealing phenotype associated with an early increase of epidermal cells producing type 2 cytokines [96]. To date, saliva-mediated enhancing effect has also been shown for other *Leishmania*–sand fly combinations; e.g., *Lu*. *longipalpis*–*L*. *braziliensis* [97], *Lu*. *longipalpis*–*L*. *amazonensis* [95], *Lu*. *longipalpis*–*L*. *chagasi* [15], *Lu*. *longipalpis*–*L*. *mexicana* [97], *Lu*. *whitmani*–*L*. *braziliensis* [98], and *P*. *duboscqi*–*L*. *major* [99]. More important is that enhancing effect is unique to sand fly saliva. Saliva from *Anopheles* *aegypti*, *Rhodnius prolixus*, or *Ixodes scapularis* did not enhance *L*. *major* infectivity in mice [94]. The sand fly saliva enhancing effect on lesion size and amount of parasites can be associated with the immunomodulatory properties as discussed previously (see section Properties of sand fly saliva).

### Protective potential of saliva and individual salivary molecules

**Fig 2:**
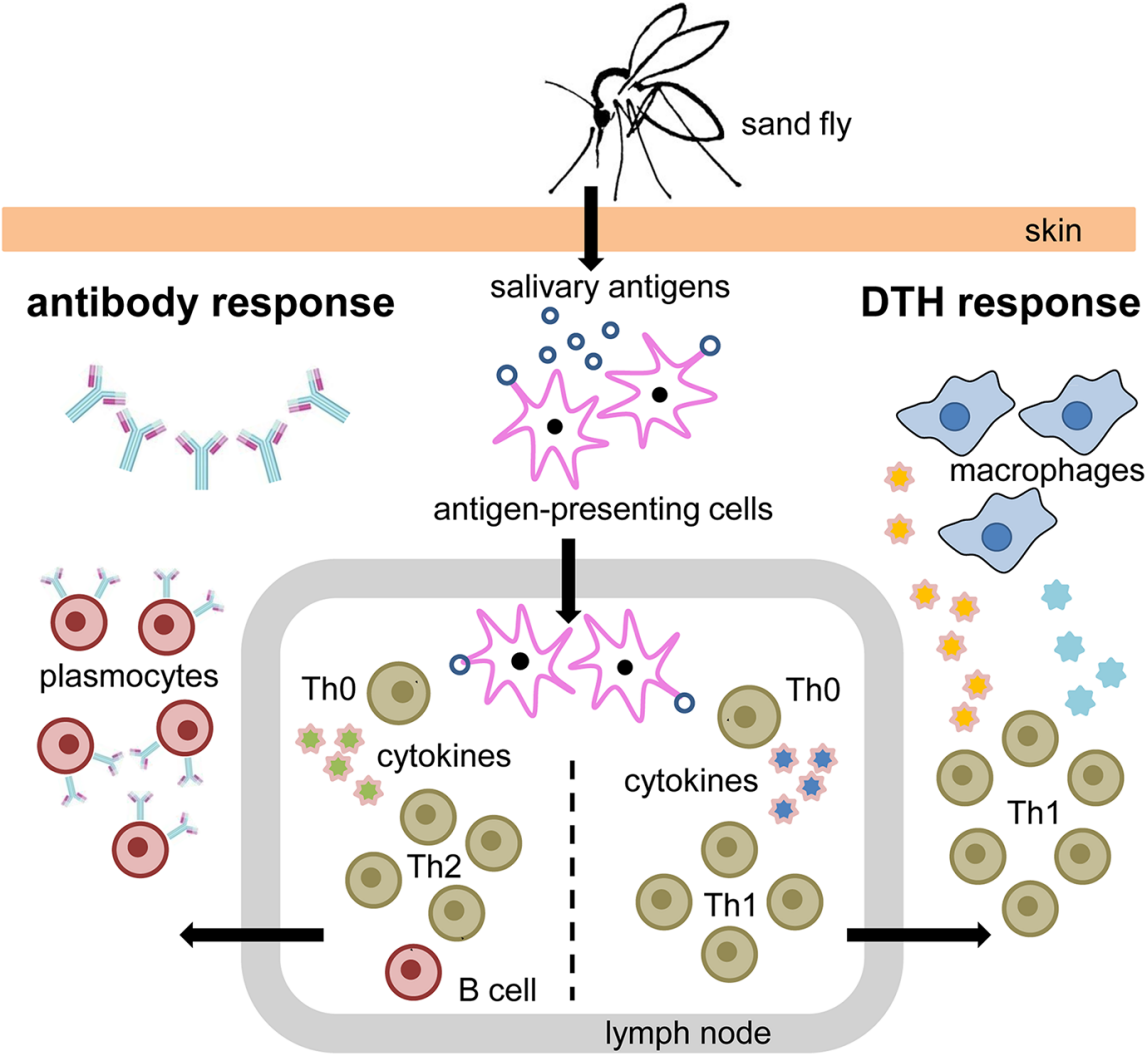
Hypothetical model depicting the immune response in a host repeatedly exposed to sand fly bites. Examples of main cytokines involved in this model are as follows: interleukin 12 (IL-12) (blue and yellow stars with pink rims), interleukin 4 (IL-4) (green stars with pink rims), interferon gamma (IFN-γ) (blue stars without rims). Abbreviations: DTH, delayed type hypersenzitivity; Th, T helper cell.

Conversely, mice repeatedly exposed to SGH or to bites of uninfected sand flies were protected against *Leishmania* infection [96, 100]. The reason is that many of the salivary components are able to induce specific immunity—both cellular and humoral, as shown in Fig 2. Therefore, the protective immunity was hypothetized to be mediated by neutralizing antibodies or delayed-type hypersensitivity (DTH) reaction at the bite site formed by a cellular influx as a response to salivary antigens [100, 101]. Although both possibilities are not mutually exclusive, later studies proved that the protection is due to DTH reaction and enhanced IFN- γ/IL-12 production [96, 100, 102]. This was further confirmed by the experiments conducted on B lymphocytes–deficient mice, which were also protected after vaccination with saliva-derived plasmid and challenged with *L*. *major* plus SGH of *P*. *papatasi* [102]. The feeding site may be changed by the presence of DTH and inflammatory cytokines elicited by sand fly salivary antigens, which create an inhospitable environment for *Leishmania* parasites [101]. As a bystander effect, this saliva-elicited immunity may even induce protection to *Leishmania* parasites (reviewed in [103]).

In laboratory settings, protection against leishmaniasis caused by *L*. *major*, *L*. *amazonensis*, and *L*. *braziliensis* due to anti-saliva cellular immunity was well described in rodent models (reviewed in [104]). Protection was elicited by both injection of *P*. *papatasi* [94], *Lu*. *longipalpis* [50, 105, 106], and *Lu*. *whitmani* SGH [107] and by exposure to *P*. *papatasi* [100] and *P*. *duboscqi* bites [99]. The protective effect of saliva was demonstrated by a smaller lesion size correlated with a decrease in parasite burden [50, 96, 99, 100, 105–107]. Moreover, pre-exposure to SGH/saliva of sand flies shifted immune response toward Th1 responsiveness characterized by increased IFN-γ and IL-12 production [50, 100] or by higher IFN-γ /IL-4 ratio [96] compared with the nonimmunized group. This response may activate infected macrophages, leading to killing of parasites during the early phase of infection, and may also promote a faster *Leishmania*-specific T helper cell type 1 response. On the other hand, type 2 cytokines such as IL-4 [96], IL-10, and TGF-β [106] were reduced in pre-exposed mice. However, in some experimental models or applied exposure schemes, the protective effect of pre-exposure to sand fly saliva or SGH was not pronounced [99, 108]. Exposure to *Lu*. *intermedia* SGH shifted the immune response to an unprotective Th2 type in BALB/c mice [108]. In fact, SGH-immunized mice developed larger lesions that prevailed for a longer period when compared with phosphate-buffered saline–inoculated mice [108].

In parallel to the demonstration that immunization with whole saliva or SGH induces protection against leishmaniasis, several works later demonstrated this same effect with individual salivary molecules of *Lu*. *longipalpis* saliva [50, 81, 109]. CBA mice injected with synthetic maxadilan were partly protected against challenge with *L*. *major* plus SGH from *Lu*. *longipalpis* [81]. Cutaneous lesions were several-fold smaller, healing by day 50 of infection, and parasite burdens were reduced in a vaccinated group. Simultaneously, addition of maxadilan to lymph node cells *in vitro* caused a release of IFN-γ and NO [81]. DNA plasmid coding for LJM19, belonging to the odorant-binding protein group, protected hamsters against infection of *L*. *infantum* mixed with SGH of *Lu*. *longipalpis* [109]. The protection was demonstrated by increased IFN-γ/TGF-β ratio and iNOS expression in the spleen and liver till 5 months post infection when compared with the control group [109]. Immunization with the YRP LJM11 or with plasmid coding for LJM11 protected mice against *L*. *major* infection [50, 110]. The increased production of IFN-γ in splenocytes after stimulation with LJM11 showed that immunity to this protein is Th1 based, which was reflected in a smaller lesion size and lower parasite burden [50]. This long-lasting immunity resulted in protection against *L*. *major* and was observed when parasites were inoculated into hosts by needle injection or when transmitted by vector bites [110].

It was shown that immunization of the host with individual salivary molecules may have diverse effects on *Leishmania* infection, contrary to whole saliva. Oliveira et al. showed that although the immunization of mice with *P*. *papatasi* SGH protected mice from *L*. *major* infection [96], immunization with PpSP44 salivary protein from this species enhanced infection caused by the same parasite. The protective outcome of infection caused by SGH exposure and the contrasting outcome caused by PpSP44 was associated with an anti-*Leishmania* Th1 and Th2 immune response, respectively [111]. In the model of *Lu*. *intermedia*–BALB/c–*L*. *braziliensis*, the plasmid coding for a Linb-11 protein was shown as a potent inducer of a cellular immune response conferring protection against *L*. *braziliensis* infection [38], contrary to the exacerbating effect of whole saliva [108].

Protection caused by salivary proteins was also described for Old World species *P*. *papatasi* and *P*. *duboscqi* [102, 112]. Vaccination with PpSP15-like protein isolated from *P*. *papatasi* affected disease progression caused by *L*. *major* in mice; lesion size and parasite load were significantly smaller compared with controls [102]. Nonhuman primates (rhesus macaques) immunized by the homologue of the aforementioned PpSP15-like protein isolated from *P*. *duboscqi* (PdSP15) were protected against *L*. *major* transmitted by infected sand fly bites [112]. Protection correlated with an early appearance of *Leishmania*-specific CD4+ IFN-γ + lymphocytes, which was reflected in reduced parasite burden compared to controls. Moreover, the immunogenicity of recombinant PdSP15 was tested in inhabitants living in the endemic area of Mali. The ability of SP15 to recall a pro-inflammatory response in humans naturally exposed to *P*. *duboscqi* bites was shown [112]. When human peripheral blood mononuclear cells were stimulated by SGH or recombinant PdSP15, significantly higher levels of IFN-γ, IL-10, and interleukin 17 (IL-17) were produced, compared to the medium. Actually, rSP15 was able to induce release of IFN-γ to a similar degree as the whole SGH, inferring rPdSP15 as a potent Th1-inducing salivary protein in humans and therefore a promising vaccine candidate against human cutaneous leishmaniasis [112].

### Cross-protective potential of saliva and individual salivary molecules

Sand fly vectors differ in composition of the saliva (reviewed in [46]), and the protection elicited by salivary proteins was shown to be species specific [105]. *Lu*. *longipalpis* saliva did not mediate cross protection against the *L*. *amazonensis* challenged together with saliva of phylogenetically distant species *P*. *papatasi* and *P*. *sergenti* [105]. Nevertheless, it was suggested that interspecies differences in the SGH protein components could correspond with the phylogenetic position of individual species [13, 32, 36], and the saliva-based vaccine could therefore be theoretically cross protective between phylogenetically related vector species with more conserved salivary proteins and thereafter applicable in more endemic foci.

In our work, we demonstrated for the first time the cross protection against *L*. *major* caused by salivary antigens of 2 closely-related *Phlebotomus* species [113]. Two groups of mice exposed to bites of *P*. *papatasi* and 2 nonimmunized groups were infected with *L*. *major* along with either *P*. *papatasi* or *P*. *duboscqi* SGH. The similarity of saliva between *P*. *duboscqi* and *P*. *papatasi* [24, 35], both proven vectors of *L*. *major* belonging to the subgenus *Phlebotomus* [114], occurring sympatrically in some areas (reviewed in [6]), probably caused the cross-protective effect. This was reflected by significantly smaller ear-lesion sizes, which corresponded to lower numbers of *Leishmania* parasites in the draining lymph node, with trends towards lower numbers of parasites also in the inoculated ear when compare with controls [113]. The cross-protective effect was also demonstrated between the *Lutzomyia* species *Lu*. *longipalpis* and *Lu*. *intermedia*, vectors of *L*. *braziliensis* [106] possessing similar salivary profiles with bands migrating at similar molecular weight [108]. Golden hamsters immunized with *Lu*. *longipalpis* SGH or with a DNA plasmid coding for the LJM19 salivary protein were protected against *L*. *braziliensis* infection in the presence of *Lu*. *intermedia* saliva, as demonstrated by reduced numbers of parasites in the inoculated ears and in the draining lymph nodes [106].

## Antibody response to sand fly saliva

Specific antibodies have been characterized after sand fly bites or injection of saliva in humans and several animal models in laboratory settings as well as in endemic areas (reviewed in [104, 115]).

### Characterization, kinetics, and specificity of anti-saliva antibodies

In mice, repeated exposure to sand fly bites or SGH resulted in increased level of anti-saliva IgG antibodies represented mainly by the IgG1 subclass [37, 91, 108, 116, 117]. In sera of immunized dogs, a significant increase of anti-saliva IgG and IgE antibodies was observed after exposure to *Lu*. *longipalpis*. However, only IgG (and IgG2 subclass) correlated with sand fly exposure intensity [118, 119]. Anti-saliva IgG and IgG2 were observed also in sera from dogs exposed to *P*. *perniciosus* bites [120]. Individuals living in areas endemic for *Lu*. *longipalpis* or volunteers exposed to uninfected laboratory-reared females of *Lu*. *longipalpis* developed predominantly IgG1 and IgE anti-saliva antibodies [121, 122]. On the other hand, antibody response to the saliva of *P*. *papatasi* in children living in Tunisia was prominently of IgG4 isotype and at a lesser extent of the IgG2 and IgG1 subclasses [123]. The humoral immune response to *Lu*. *intermedia* was also characterized by the presence of IgG1 and IgG4 in naturally exposed individuals, in the absence of IgE [124]. These results show that in humans, antibody response to sand fly saliva may differ among genetically variable host populations, and it could also be influenced by sand fly species.

In endemic areas, sand fly population fluctuates seasonally (reviewed in [6]), which may influence host anti-saliva antibody response. There are several studies focusing on the long-term kinetics of anti-saliva antibodies in mice [116, 117], dogs [118, 120], humans [122, 125], or rabbits [117]. In humans repeatedly bitten by *P*. *argentipes*, specific antibodies significantly declined within 30 days of a sand fly–free period, although they have persisted in low levels for 5 months after the last sand flies exposure [125]. An increased specific anti-saliva antibody response was still detected in dogs and mice after 6 months biting-free period of *Lu*. *longipalpis* and *P*. *papatasi*, respectively [116, 118]. However, a rapid antibody decrease in canine sera was observed within 1 week after the last *P*. *perniciosus* exposure [120], reflecting changes in the vector-exposure intensity. Importantly, after the 1-year or 6-month biting-free period, further reexposure with *Lu*. *longipalpis* or *P*. *argentipes* bites, respectively, caused significant increase of antibody levels in humans [122, 125], which indicates an antibody memory response to saliva for both sand fly species. An effective recall immune response was observed also in mice and rabbits bitten by *P*. *perniciosus* [116, 117].

Antibody response elicited by sand fly salivary proteins was shown to be species specific, e.g., [14, 105, 108, 126–129]. Mice exposed individually to *P*. *papatasi*, *P*. *sergenti*, or *Lu*. *longipalpis* produced antibodies specific to the respective species [105]. Sand fly species-specific salivary antigen was also observed among *P*. *perniciosus*, *P*. *halepensis*, and *P*. *papatasi* [14]. Even though the salivary profiles of *Lu*. *longipalpis* and *Lu*. *intermedia* are similar, their antigenic properties seemed to be different [108]; serum samples from mice immunized with SGS of *Lu*. *intermedia* recognized only 1 *Lu*. *longipalpis* SGS protein of about 45 kDa [108]. Moreover, the antigenicity of salivary proteins is also host-species specific [126, 130, 131]. Several differences in the recognition pattern were observed between hamster and murine anti–*P*. *perniciosus* antibodies. While YRPs and apyrases were recognized by both rodents, D7-related proteins reacted only with hamster antibodies [130]. Interestingly, some *P*. *perniciosus* salivary antigens were specifically recognized solely by hare or rabbit anti–*P*. *perniciosus* antibodies, while some salivary antigens were common to those 2 host species, despite the individual pattern in the intensity of reaction [131]. Main salivary bands identified in *P*. *papatasi* and *P*. *sergenti* saliva reacted with mouse as well as with human sera; nevertheless, differences were observed in the intensity of reaction [126]. The comprehensive summary of these immunogenous salivary proteins recognized by the broad spectrum of bitten hosts is shown in Table 2.

**Table 2:** The most antigenic salivary protein families recognized by sera of repeatedly bitten hosts.

Salivary protein	Sand fly species	Host species	Reference
**YRP**	*Lutzomyia longipalpis*	human	Gomes et al. 2002 [121]
		mice	Rohousova et al. 2005 [126]
		dogs	Bahia et al. 2007 [148]
		dogs	Hostomska et al. 2008 [118]
		foxes, dogs	Gomes et al. 2007 [127]
		chickens	Soares et al. 2013 [142]
	*Phlebotomus arabicus*	mice	Hostomska et al. 2009 [34]
	*P*. *papatasi*	mice	Rohousova et al. 2005 [126]
		mice	Vlkova et al. 2012 [116]
		human	Marzouki et al. 2011 [123]
	*P*. *perniciosus*	mice, hamsters	Martin-Martin et al. 2012 [130]
		mice, rabbits	Martin-Martin et al. 2015 [117]
		hares, rabbits	Martin-Martin et al. 2014 [131]
		dogs	Vlkova et al. 2011 [120]
	*P*. *tobbi*	rabbit	Rohousova et al. 2012 [36]
	*P*. *orientalis*	dogs	Sima et al. 2016 [144]
**apyrase**	*Lu*. *longipalpis*	human	Gomes et al. 2002 [121]
		dogs	Hostomska et al. 2008 [118]
		mice	Rohousova et al. 2005 [126]
	*P*. *arabicus*	mice	Hostomska et al. 2009 [34]
	*P*. *papatasi*	human	Rohousova et al. 2005 [126]
		mice	Vlkova et al. 2012 [116]
		human	Marzouki et al. 2011 [123]
	*P*. *perniciosus*	mice	Martin-Martin et al. 2012 [130]
		mice, rabbits	Martin-Martin et al. 2015 [117]
		hares, rabbits	Martin-Martin et al. 2014 [131]
		dogs	Vlkova et al. 2011 [120]
	*P*. *tobbi*	rabbit	Rohousova et al. 2012 [36]
	*P*. *orientalis*	dogs	Sima et al. 2016 [144]
**D7-related**	*Lu*. *longipalpis*	dogs	Bahia et al. 2007 [148]
		dogs	Hostomska et al. 2008 [118]
	*P*. *papatasi*	human	Rohousova et al. 2005 [126]
		mice	Vlkova et al. 2012 [116]
		human	Marzouki et al. 2011 [123]
	*P*. *perniciosus*	dogs	Vlkova et al. 2011 [120]
		hares, rabbits	Martin-Martin et al. 2014 [131]
		hamsters	Martin-Martin et al. 2012 [130]
		mice, rabbits	Martin-Martin et al. 2015 [117]
	*P*. *tobbi*	rabbit	Rohousova et al. 2012 [36]
	*P*. *orientalis*	dogs	Sima et al. 2016 [144]
**antigen-5**	*P*. *papatasi*	mice	Vlkova et al. 2012 [116]
	*P*. *perniciosus*	dogs	Vlkova et al. 2011 [120]
	*P*. *tobbi*	rabbit	Rohousova et al. 2012 [36]
	*P*. *orientalis*	dogs	Sima et al. 2016 [144]
**SP-15**	*P*. *papatasi*	mice	Vlkova et al. 2012 [116]
		human	Marzouki et al. 2011 [123]
	*P*. *perniciosus*	dogs	Vlkova et al. 2011 [120]
		mice, rabbits	Martin-Martin et al. 2015 [117]
	*P*. *tobbi*	rabbit	Rohousova et al. 2012 [36]

**Abbreviation:** YRP, yellow-related protein.

### Multiple uses of anti-saliva antibody response

#### Anti-saliva antibodies as a marker of exposure

Because anti-saliva antibodies correlate well with the intensity of exposure [116–118, 120], they can be used in epidemiological studies, e.g., to measure the effectiveness of vector-control programmes and to design better strategies for the control of leishmaniasis in the spreading foci [125, 132]. To this date, a significant correlation between levels of specific IgG anti-saliva antibodies and intensity of exposure was documented in mice [116, 117], dogs [118, 120], and humans [125] as well as in leporids [117].

#### Anti-saliva antibodies as a marker of risk for *Leishmania* transmission

The higher titer of anti-saliva antibodies suggests more frequent contact with sand flies, thus increasing probability to encounter infected bites [108, 123, 126]. Anti-saliva antibodies specific to *P*. *sergenti*, *Lu*. *intermedia*, or *P*. *papatasi* were utilized as a risk marker of cutaneous leishmaniasis [108, 123, 126] and moreover associated with the disease development [108, 123, 124]. However, this association was not proven in the *Lu*. *whitmani*–*L*. *braziliensis* model [107]. These results suggest that, although salivary contents may be similar between *Lu*. *intermedia* and *Lu*. *whitmani*, the vectors of *L*. *braziliensis*, there are immunodominant salivary molecules that drive different outcomes following natural exposure in endemic settings.

On the other hand, a different scenario seems to be valid for vectors of *Leishmania* causing visceral leishmaniasis (reviewed in [104, 115]). In this case, the co-occurrence of anti-saliva antibodies and anti-*Leishmania* DTH reaction was observed in humans [121, 122, 133, 134], suggesting that immune response against SGS correlates with a protective response against leishmaniasis. Moreover, individuals who did not recognize salivary proteins developed anti-*Leishmania* antibodies generally associated with disease progress [121, 135]. However, further studies are needed to validate this hypothesis.

#### Anti-saliva antibodies as an indicator of putative reservoirs

Concurrently, the use of anti-saliva antibodies is a novel approach that can indicate an important blood source for sand flies or parasite hosts and putative reservoirs. The existence of a sylvatic cycle independent of the previously well-known domestic cycle was confirmed by using this approach in Brazil [127] and Spain [131]. In both cases, dogs were expected to be the main reservoir hosts of *L*. *chagasi* and *L*. *infantum*, respectively (reviewed in [136]). In Brazil, sylvatic cycle of *L*. *chagasi* has been revealed among wild foxes (*Cerdocyon thous*) [127]; high levels of anti–*Lu*. *longipalpis* SGH antibodies were found among local foxes but not among those living in regions where *Lu*. *longipalpis* is absent. Infection by *Leishmania* parasites was even detected in 3 foxes [127]. A new wild reservoir of the causative agent of visceral leishmaniasis was also confirmed in Spain [137]. Hares (*Lepus granatensis*) caught in a peri-urban green park situated southwest of Madrid showed higher anti-saliva antibody levels compared to hares from a nonendemic region, indicating frequent contact with vector bites [131]. Furthermore, these hares were able to infect *P*. *perniciosus* sand flies with *L*. *infantum*, which has been demonstrated by xenodiagnostic transmission [137]. The participation of domestic animals in the epidemiology of leishmaniasis caused by *L*. *donovani* in East Africa, an area traditionally considered to be anthroponosis, was also indicated based on widespread exposure to *P*. *orientalis* saliva. Nevertheless, the direct evidence proving animals as parasite hosts warrants further investigation [138]. Additionally, anti-saliva antibodies can be also used to monitor sand flies’ distribution in endemic regions (as mentioned in [139]).

#### Anti-saliva antibodies as an indicator of Th phenotype

Anti-saliva antibody response could be also used as a marker of cell-mediated immune response. Reciprocal regulatory interaction between T-cell subsets has potent effects on B cell differentiation. In a murine model, cytokines such as IFN-γ and IL-4 secreted by distinct T-cell populations were shown to promote expression of specific Ig subtypes. While Th1 cell-derived IFN-γ stimulates IgG switch to IgG2a isotypes, Th2 cell-derived IL-4 promotes switching to IgG1 and IgE expression [140]. It was shown that sera of mice immunized with plasmids encoding the strongest inducer of DTH and antibody response (*P*. *ariasi* SP25-like protein) produced significantly higher levels of IgG1 (Th2 phenotype) antibodies when compared with the IgG2a subclass (Th1 phenotype) [37]. On the other hand, immunization with plasmids coding for *Lu*. *longipalpis* LJL143 and LJM17 triggered cellular response as well as dominant IgG2 antibodies in immunized dogs, indicating Th1 profile [119]. Thus, characterization of anti-saliva IgG subclasses might help in selecting candidate proteins for anti-*Leishmania* vaccine.

## Utilization of recombinant salivary proteins to estimate sand fly exposure

As mentioned above, the antigenic properties of sand fly saliva provide a possibility for its utilization as an indicator of close contact between a host and a sand fly. In such studies, possible cross reactivity between sympatrically occuring sand fly species or even between sand flies and other blood-feeding insects should be excluded since the cross reaction could lead to false-positive results (reviewed in [115]).

So far, in most studies, the anti-saliva antibodies have been detected using whole SGH. The advantage is that it represents the complete repertoire of secreted salivary proteins that are in native forms. However, this approach also has some limitations: closely related sand fly species have a higher probability of shared salivary antigens, and thus utilization of total saliva as antigen might hamper the identification of species-specific markers (reviewed in [115]). Another limitation for large-scale serological studies is the maintainance of the sand fly colony for salivary gland dissection, which is economically demanding, time-consuming, and requires a well-trained person. Additionally, the protein composition and quantity obtained in salivary gland extract may vary even in long-term established colonies due to differences in sand fly physiological factors such as age and diet [13, 141].

To overcome these limitations, the identification of a single, species-specific salivary protein would be beneficial (reviewed in [104, 115]) and the goal is to express salivary antigens in recombinant forms in controlled quality and large quantity for use in large epidemiological studies. Such studies have been performed so far only on 4 sand fly species: *Lu*. *longipalpis*, *P*. *papatasi*, *P*. *orientalis*, and *P*. *perniciosus* [117, 128, 129, 131, 139, 142–147].

Previous studies had already shown that anti–*Lu*. *longipalpis* antibodies in human or animal serum recognize specific salivary proteins of different molecular weight [91, 118, 121, 122, 127, 133, 142, 148]. Nine of the most antigenic salivary proteins, present in *Lu*. *longipalpis*, recognized by human, canine, or fox sera, were produced in a mammalian expression system [128]. Among them, the best candidates were LJM17 (45 kDa YRP), recognized by sera from all 3 aforementioned hosts, and LJM11 (43 kDa YRP), recognized by human and dog serum samples [128]. These recombinant proteins were further tested in a large-scale study using individuals from places endemic for visceral leishmaniasis [139]. Human sera, which recognized *Lu*. *longipalpis* SGH in ELISA, also recognized the mixture of rLJM17 and rLJM11 proteins, and the detection of seroconversion was significantly improved using this combination [139]. These 2 molecules were also used to monitor chicken exposure to phlebotomine bites in Brazil [142]; results obtained with SGH were positively correlated with those obtained with rLJM11 but not with rLJM17 [142], highlighting host-specificity of anti-saliva antibody response. Moreover, both aforementioned recombinant proteins were specifically recognized by humans exposed to *Lu*. *longipalpis* but not by individuals exposed to *Lu*. *intermedia* [128], thus showing the desired specificity.

Antibodies from humans and animals exposed to *P*. *papatasi* bites recognized mainly proteins of 42, 36, and 30 kDa [120, 123, 126]. The last one was prepared in a recombinant form in a mammalian expression system and further tested with sera of humans naturally exposed to *P*. *papatasi* in Tunisia and Saudi Arabia, areas endemic for cutaneous leishmaniasis [129, 143, 149]. A study conducted in Tunisia described rPpSP32 as the immunodominant antigen, able to act as an alternative to saliva for screening of sand fly exposure [129, 143]. Moreover, the binding of human IgG antibodies to native PpSP32 was inhibited by preincubation of serum samples with the recombinant form of PpSP32, proving similarities between the recombinant and native forms of this protein [129]. In addition, sera obtained from humans and dogs immunized by *P*. *perniciosus* bites, a species widely present in Tunisia, did not react with rPpSP32, confirming absence of cross reaction between these 2 sympatric species [129, 143].

Furthermore, the 5 major salivary antigens of *P*. *orientalis* were identified as a ParSP25-like protein, a YRP, an antigen 5-related protein, an apyrase, and a D7-related protein. They were expressed in an *E*. *coli* system and used for detection of IgG antibodies in sera of domestic animals collected in Ethiopia [144]. The ELISA tests revealed the recombinant YRP (rPorSP24) as the most universal candidate replacing whole SGH. The convincing correlation has been achieved in various host species including sheep, goats, and dogs. Moreover, the specificity of this *P*. *orientalis* recombinant antigen was proved by using murine sera experimentally exposed to sympatrically occuring species (*P*. *papatasi* and *Sergentomyia schwetzi*), as antibodies from mice bitten by each aforementioned sand fly species did not react with rPorSP24 [144].

Recent studies showed that dogs experimentally bitten by *P*. *perniciosus* recognized with the highest affinity a YRP (42 kDa), followed by 2 apyrases (38 kDa, 33 kDa) and an antigen 5 protein (29 kDa) [120]. From the bacterially expressed salivary proteins of *P*. *perniciosus*, recombinant YRP (rSP03B) and 2 apyrases (rSP01 and rSP01B) were chosen as the best candidates for the exposure assessment in mice and dogs experimentally bitten with *P*. *perniciosus* females [145]. The antibody response targeting these 3 recombinant proteins correlated well with the anti-SGH antibody response not only in experimentally exposed hosts [145] but also in naturally bitten dogs and hares [131]. *P*. *perniciosus* recombinant YRP rSP03B showed the best correlation scores for hares and rabbits compared with SGH. Moreover, it seems to be the best marker of canine exposure because it presents the lowest data dispersion [131]. Recently, recombinant apyrase (rSP01B) and D7-related protein (rSP04) from *P*. *perniciosus* were tested with serum samples obtained from laboratory-exposed mice [117]. While anti-saliva antibodies showed similar reactivity to rSP01B and SGH, they exhibited highly variable reactivity to rSP04. Therefore, rSP01B seems to be the best candidate for marker of *P*. *perniciosus* exposure in this sand fly–host model [117].

Although all aforementioned publications confirm the advantages of employing recombinant salivary proteins for field studies, most of these experiments were performed on small sets of samples [129, 131, 142], their correlation with complete SGH was not convincing [139, 142, 143], or the results were never associated with the infection status of the hosts.

In a longitudinal field study, rSP03B was confirmed to be a valid alternative to SGH of *P*. *perniciosus* [146]. The curve depicting sand fly occurence over biennial monitoring season showed a similar pattern comparing anti-SGH and anti-rSP03B IgG antibodies. Levels of antibodies against both antigens were higher during summer months and declined during winter months, closely reflecting *P*. *perniciosus* seasonality in Italy [150]. Nevertheless, in conflict with results indicating a significant positive association between anti–*P*. *perniciosus* saliva antibodies and active *L*. *infantum* infection, we did not find any association between antibodies against rSP03B protein and active canine *Leishmania* infection, and therefore, the use of recombinant proteins as risk markers for infection will need more investigation [146]. In the end, we confirmed regionally universal use of rSP03B as a marker of sand fly exposure on 550 canine serum samples originating from distant localities of *P*. *perniciosus* occurrence [147]. In addition, the binding of canine IgG antibodies to native SP03B was inhibited by preincubation of serum samples with the recombinant form of this protein, showing shared antigenic epitopes between the recombinant and native forms of this YRP [147].

The best-performing recombinant proteins confirmed as markers of exposure to sand fly bites in experimental models as well as in field settings are summarized in Table 3.

**Table 3:** The best recombinant salivary candidates as antigens for detection of anti-saliva antibodies.

Recombinant protein	Protein family	Sand fly species	Host species	Reference
LJM17	YRP	*Lutzomyia longipalpis*	dog, fox, human	Teixeira et al. 2010 [128]; Souza et al. 2010 [139]
LJM11	YRP	*Lu*. *longipalpis*	human, dog, chicken	Teixeira et al. 2010 [128]; Soares et al. 2013 [142]
LJM17+LJM11	YRP	*Lu*. *longipalpis*	human	Souza et al. 2010 [139]
rPpSP32	SP32-like	*Phlebotomus papatasi*	human	Marzouki et al. 2012 [129], 2015 [143]; Mondragon-Shem et al. 2015 [149]
rPorSP24	YRP	*P*. *orientalis*	sheep, goat, dog	Sima et al. 2016 [144]
rSP03B	YRP	*P*. *perniciosus*	mouse, dog, hare, rabbit	Drahota et al. 2014 [145]; Martin-Martin et al. 2014 [131]; Kostalova et al. 2015 [146]; Kostalova et al. 2016 [147]
rSP01	apyrase	*P*. *perniciosus*	mouse, dog	Drahota et al. 2014 [145]
rSP01B	apyrase	*P*. *perniciosus*	mouse, dog, hare, rabbit	Drahota et al. 2014 [145]; Martin-Martin et al. 2014 [131]; Martin-Martin et al. 2015 [117]

**Abbreviation:** YRP, yellow-related protein.

Futher works could focus on predictions and identifications of putative B cell salivary epitopes by *in silico* approaches, with subsequent designing of synthetic peptides serving as the immunoassay antigens and markers of sand fly exposure. Synthetic peptides were already used to assess human exposure to tsetse flies or mosquitoes, e.g., [151–156], but they have not yet been applied on sand flies. Studies of antibodies’ reactivity to a single epitope could provide a better specificity and sensitivity when compared to whole SGH. Moreover, synthetic peptides have several advantages when compared with salivary recombinant proteins. While the production and storage of recombinant proteins are often problematic and require good facilities that limit their use in many contexts, peptides are more easily produced and shipped, as they can be stored lyophilized.

**Key learning points**

- Sand fly saliva is composed of pharmacologically active components with antihemostatic, anti-inflammatory, and immunomodulatory properties, which facilitate blood-meal intake.
- *Leishmania* parasites are coinoculated with saliva into the blood-feeding site and they can benefit from saliva-altered local immune reaction. In naive hosts, sand fly saliva exacerbates the infection by *Leishmania* sp., causing "enhancing effect," reflected by larger lesions and higher parasite numbers.
- Conversely, hosts repeatedly exposed to bites of uninfected sand flies or immunized by certain salivary proteins were protected against *Leishmania* infection. Therefore, sand fly salivary antigens are currently used to develop a vaccine against leishmaniasis.
- Hosts repeatedly bitten by sand flies develop specific anti-saliva antibodies. Levels of anti-saliva IgG reflect the intensity of exposure to sand flies and thus can be used in epidemiological studies, e.g., to measure the effectiveness of vector-control campaigns or as a marker of risk for *Leishmania* transmission.

**Top 5 papers**

1. Andrade BB, Teixeira CR. Biomarkers for exposure to sand flies bites as tools to aid control of leishmaniasis. Front Immunol. 2012;3:121. 10.3389/fimmu.2012.00121. 22661974; PubMed Central PMCID: PMCPMC3356838.
2. Kamhawi S, Belkaid Y, Modi G, Rowton E, Sacks D. Protection against cutaneous leishmaniasis resulting from bites of uninfected sand flies. Science. 2000;290(5495):1351–4. 10.1126/science.290.5495.1351. WOS:000165379800047.
3. Oliveira F, Lawyer PG, Kamhawi S, Valenzuela JG. Immunity to distinct sand fly salivary proteins primes the anti-*Leishmania* immune response towards protection or exacerbation of disease. PLoS Negl Trop Dis. 2008;2(4). e22610.1371/journal.pntd.0000226. WOS:000261806700009.
4. Ribeiro JM, Mans BJ, Arcà B. An insight into the sialome of blood-feeding Nematocera. Insect Biochem Mol Biol. 2010;40(11):767–84. S0965-1748(10)00170-0 [pii] 10.1016/j.ibmb.2010.08.002. 20728537; PubMed Central PMCID: PMCPMC2950210.
5. Teixeira C, Gomes R, Collin N, Reynoso D, Jochim R, Oliveira F, et al. Discovery of markers of exposure specific to bites of *Lutzomyia longipalpis*, the vector of *Leishmania infantum chagasi* in Latin America. PLoS Negl Trop Dis. 2010;4(3):e638. 10.1371/journal.pntd.0000638. 20351786; PubMed Central PMCID: PMCPMC2843637.
